# Data on the utilization of paraneoplastic syndrome autoantibody testing at an academic medical center

**DOI:** 10.1016/j.dib.2021.107578

**Published:** 2021-11-18

**Authors:** Matthew D. Krasowski, Anna Dolezal, Bryan W. Steussy, Michael P. Gailey, Benjamin W. Darbro

**Affiliations:** a Department of Pathology, University of Iowa Hospitals and Clinics, 200 Hawkins Drive C-671 GH, Iowa City, IA 52242, USA; b Central Oregon Pathology Consultants, Bend, OR 97701, USA; c Pathology Medical Services of Siouxland, PC, UnityPoint Health-St. Luke's, Sioux City, IA 51104, USA; d Vista Pathology Laboratory, Medford, OR 97501, USA; e Stead Family Department of Pediatrics, University of Iowa Stead Family Children's Hospital, Iowa City, IA 52242, USA

**Keywords:** Anti-N-methyl-D-aspartate receptor encephalitis, Autoantibodies, Paraneoplastic syndromes, Utilization review, Voltage-gated calcium channels, Voltage-gated potassium channels

## Abstract

Paraneoplastic syndromes are rare conditions associated with characteristic autoantibodies produced by malignancy, although similar autoantibodies and clinical presentations may occur in the absence of any neoplasm. Testing for paraneoplastic syndromes often involves panels of autoantibody assays. While autoantibody testing may reveal or confirm actionable clinical diagnoses, inappropriate utilization of testing may be low yield and further lead to false positives that may confuse the clinical picture. There is thus opportunity to improve patient care by analyzing patterns of paraneoplastic autoantibody test utilization. The data in this article provides results from detailed retrospective review of patients tested by 7 autoantibody tests or test panels offered by two large reference laboratories in the United States. The data include 1,446 tests performed on 1,338 unique patients at an academic medical center. For all results, detailed chart review revealed main category of presenting symptoms, patient location at time of testing (either inpatient or outpatient), sex, age, whether cancer was present at the time of testing or later detected, and the specific results of the testing. The data are summarized by category of testing and specific autoantibodies.

## Specifications Table

**Table:** 

Subject	Medicine and Dentistry
Specific subject area	Pathology and Medical Technology
Type of data	Figures Tables Supplemental files
How data were acquired	Retrospective chart and data review from laboratory analysis performed at an academic medical center central clinical laboratory were obtained via tools within the electronic medical record.
Data format	Raw and Analyzed
Parameters for data collection	Retrospective data on all paraneoplastic autoantibody testing were obtained from the electronic medical record (Epic, Inc.) covering the time period from December 1, 2008 through November 30, 2018. Detailed chart review was performed for all cases. The project had approval from the University of Iowa Institutional Review Board.
Description of data collection	There were a total of 1,446 paraneoplastic autoantibody tests performed on 1,338 unique patients during the retrospective analysis period (with some patients receiving more than one type of test). The data collection contained results of the following laboratory testing, consisting of either panels of testing or discrete components offered as stand-alone tests: paraneoplastic autoantibody panel in serum (Mayo Clinic Laboratories), paraneoplastic autoantibody panel in cerebrospinal fluid (CSF; Mayo), paraneoplastic autoantibody panel in serum (ARUP Laboratories), *N*-methyl-*D*-aspartate (NMDA) receptor autoantibodies in serum (ARUP), NMDA receptor autoantibodies in CSF (ARUP), voltage-gated calcium channel autoantibodies in serum (ARUP), and voltage-gated potassium channel autoantibodies in serum (ARUP).
Data source location	University of Iowa Hospitals and Clinics, Iowa City, Iowa, United States of America
Data accessibility	Three tables and one figure are included within the paper. 7 Supplementary files are deposited in Mendeley: Data identification number: https://doi.org/10.17632/ydskhmmmz4.2 Direct URL to data: https://data.mendeley.com/datasets/ydskhmmmz4/2

## Value of the Data

• The data provided are of value as paraneoplastic autoantibody testing may be over-utilized, potentially leading to excess costs and downstream impact on patients.
• Clinicians, other researchers, or personnel in clinical laboratories might find this data useful as a reference for comparison.
• Our data set would serve as a starting point for researchers interested in future investigations investigating utilization of paraneoplastic autoantibody testing.
• The data provide information on paraneoplastic autoantibody testing at an academic medical center over a decade.
• The data provide information for 1,446 paraneoplastic autoantibody tests performed on 1,338 unique patients.

## 1 Data Description

Paraneoplastic syndromes are rare conditions associated with autoantibodies that are classically produced by malignancies but may also occur in the absence of any neoplasm [1], [2], [3]. Testing for paraneoplastic syndromes often involves panels of autoantibody assays [4], [5], [6], [7]. The appropriate utilization of paraneoplastic autoantibody panels has been debated, with multiple studies showing relatively low yield of diagnostic testing and a high rate of false positives [8], [9], [10], [11], [12], [13]. A primary factor may be testing in populations without appropriate clinical phenotype and context for paraneoplastic syndromes, providing opportunity for education to promote improved utilization of this area of laboratory testing [9,[14], [15], [16], [17]. There is thus opportunity for collaboration between pathology and clinical services that commonly order paraneoplastic and autoimmune encephalitis autoantibody testing in the design of algorithms and guidelines for testing [4].

This retrospective analysis study includes data (including detailed chart review) on 1,446 samples originating from 1,338 unique patients who had paraneoplastic autoantibody testing ordered at an academic medical center. The laboratory testing data includes panels of serum paraneoplastic autoantibody panels offered by two reference laboratories in the United States (Mayo Clinic Laboratories, Rochester, MN; ARUP Laboratories, Salt Lake City, UT) and a CSF paraneoplastic autoantibody panel by Mayo Clinic Laboratories. ARUP Laboratories additionally offers stand-alone assays for *N*-methyl-*D*-aspartate (NMDA) receptor autoantibodies in either CSF or serum as well as separate assays for the P/Q-type voltage-gated calcium channel (VGCC) autoantibodies or voltage-gated potassium channel (VGKC) autoantibodies in serum.

Table 1 shows the laboratory panels analyzed in the present study and their constituent antibody tests. The antibody types are divided into those directed towards intracellular antigens, neuronal surface antigens, and neuromuscular antigens. The paraneoplastic serum panel at Mayo Clinic Laboratories is more extensive than that offered at ARUP Laboratories. The Mayo paraneoplastic CSF panel overlaps somewhat with the Mayo serum panel but does not include some antibodies tested for by the serum panel and also has some antibodies not included in the serum panel. These do not represent all of the paraneoplastic testing available at ARUP Laboratories and Mayo Clinic Laboratories, but rather those ordered at our medical center. Ordering options for panels and standalone assays have changed over time. Table 2 summarizes the total number of tests and unique patients for the various panels or stand-alone laboratory tests analyzed in the present study. There was a total of 1446 paraneoplastic autoantibody tests performed on 1338 unique patients, with some patients receiving more than one type of test (e.g., both Mayo serum and CSF paraneoplastic panels). A footnote to Table 2 also notes a small number of patients who had indeterminate results on testing if the reference laboratory had that category as a possible result based on quantitative signal. Table 3 summarizes the specific antibodies detected in the present study, which testing included the positive antibody (either a panel or stand-alone test), and the number of malignancies found.

**Table 1:** Laboratory panels and their constituent antibody tests.

Antibody type	Antibodies^1^	ARUP Laboratories paraneoplastic serum panel	Mayo Clinic Laboratories paraneoplastic serum panel	Mayo Clinic Laboratories paraneoplastic CSF panel^1^	Standalone assay at ARUP Laboratories
Intracellular antigens	AGNA-1		Yes	Yes	
	Amphiphysin	Yes^1^	Yes	Yes	
	ANNA-1 (Hu)	Yes	Yes	Yes	
	ANNA-2 (Ri)	Yes	Yes	Yes	
	ANNA-3		Yes	Yes	
	CRMP-5 (CV2)	Yes^1^	Yes	Yes	
	GAD65		Yes	Yes	
	Ma/Ta		Yes		
	PCCA-1 (Yo)	Yes	Yes	Yes	
	PCCA-2		Yes	Yes	
	PCCA-Tr		Yes	Yes	
Neuronal surface antigens	AMPA receptor			Yes	
	GABA receptor			Yes	
	NMDA receptor			Yes	Yes
Neuromuscular antigens	AChR binding		Yes		
	AChR ganglionic		Yes		
	AChR modulating		Yes		
	AQP4			Yes	
	VGCC-N		Yes		
	VGCC-PQ		Yes		Yes
	VGKC		Yes	Yes	Yes
	Striational		Yes		

1 *Abbreviations:* AChR, acetylcholine receptor; AGNA, anti-glial nuclear antibody; AMPA, α-amino-3-hydroxy-5-methyl-4-isoxazolepropionic acid; ANNA, antineuronal antibody; AQP4, aquaporin-4; CRMP-5, collapsing response-mediator protein-5; CSF, cerebrospinal fluid; GABA, gamma-aminobutyric acid; GAD, glutamic acid decarboxylase; NMDA, *N*-methyl-*D*-aspartate; PCCA, Purkinje cell cytoplasmic antibody; VGCC, voltage-gated calcium channel; VGKC, voltage-gated potassium channel. The ARUP serum paraneoplastic panel was updated 12/22/2016 to include amphiphysin and CV2.1 antibodies.

**Table 2:** Total number of tests.

Reference Laboratory	Panel or Test	# of Unique Patients (Females/ Males)	# of Total Tests (Including Repeats)	Mean/ Median Age (yrs)	1 or More Antibodies Detected (Unique Patients)^2^	Total Number of Patients with a Malignancy (% of Unique Patients)
Mayo	Paraneoplastic panel, serum	225 / 258	487	58.0 / 59.1	76 / 483 (15.7%)	37 (7.7%)
Mayo	Paraneoplastic panel, CSF^1^	137 / 139	280	55.4 / 59.5	6 / 276 (2.2%)	19 (6.9%)
ARUP	NMDA, CSF	84 / 51	142	45.6 / 49.3	7 / 135 (5.2%)^2^	4 (3.0%)
ARUP	NMDA, serum	86 / 58	150	45.3 / 51.5	5 / 144 (3.5%)	7 (4.9%)
ARUP	VGCC, serum	26 /19	56	60.2 / 65.1	3 / 45 (6.7%)^2^	11 (24.4%)
ARUP	VGKC, serum	57 /50	110	55.9 / 58.4	3 / 107 (2.8%)^2^	9 (8.4%)
ARUP	Paraneoplastic panel, serum	103 / 102	215	58.7 / 62.1	3 / 205 (1.5%)	15 (7.5%)

1 *Abbreviations:* CSF, cerebrospinal fluid; NMDA, *N*-methyl-*D*-aspartate; VGCC, voltage-gated calcium channel; VGKC, voltage-gated potassium channel.

2 Some patients had more than one positive result for some components. There was one additional patient with an indeterminate result on the ARUP NMDA CSF test. Two of the patients for the ARUP VGCC serum test had both indeterminate and positive results at various timepoints. There were 12 patients with indeterminate results for the ARUP VGKC test.

**Table 3:** Antibodies detected by the panels.

Reference Laboratory	Panel or Test	Specific Antibody Detected	# of Positive Samples per Unique Patients (%)	Sex	Mean age (yrs)	Number of Unique Patients with Malignancy and Positive Test Result (% of Total Patients)
Mayo	Paraneoplastic panel, serum	AChR binding	9/ 483 (1.9%)	3 F, 6 M	64.2	0 (0.0%)
Mayo	Paraneoplastic panel, serum	AChR ganglionic	8 / 483 (1.7%)	3 F, 5 M	67.9	0 (0.0%)
Mayo	Paraneoplastic panel, serum	Striational	38 / 483 (7.9%)	21 F, 17 M	57.6	2 (0.4%)
Mayo	Paraneoplastic panel, serum	VGCC-PQ	9 / 483 (1.9%)	2 F, 7 M	59.4	2 (0.4%)
Mayo	Paraneoplastic panel, serum	VGCC-N	4 / 483 (0.8%)	1 F, 3 M	53.4	0 (0.0%)
Mayo	Paraneoplastic panel, serum	VGKC	20 / 483 (4.1%)	4 F, 16 M	64.7	4 (0.8%)
Mayo	Paraneoplastic panel, serum	CRMP-5	2 /483 (0.4%)	2 F	67.8	1 (0.2%)
Mayo	Paraneoplastic panel, serum	ANNA-1 (Hu)	2 / 483 (0.4%)	1 F, 1 M	59.6	0 (0.0%)
Mayo	Paraneoplastic panel, CSF	GAD65	1 / 276 (0.4%)	1 M	62.7	0 (0.0%)
Mayo	Paraneoplastic panel, CSF	NMDA	3 / 276 (1.1%)	3 F	36.7	0 (0.0%)
Mayo	Paraneoplastic panel, CSF	Purkinje	2 / 276 (0.7%)	1 F, 1M	66.0	2 (0.7%)
ARUP	NMDA, CSF	NMDA	7 / 135 (5.2%)	5 F, 2 M	32.0	0 (0.0%)
ARUP	NMDA, serum	NMDA	5 / 144 (3.5%)	3 F, 2M	17.5	0 (0.0%)
ARUP	VGCC, serum	VGCC	3 / 45 (6.7%)	2 F, 1M	71.6	1 (2.2%)
ARUP	VGKC, serum	VGKC	3 / 107 (2.8%)	3 F	47.4	0 (0.0%)
ARUP	Paraneoplastic panel, serum	ANNA-1 (Hu)	3 / 205 (1.5%)	2 F, 1M	66.2	2 (1.0%)

^1^Abbreviations: AChR, acetylcholine receptor; ANNA, antineuronal antibody; CRMP-5, collapsing response-mediator protein-5; CSF, cerebrospinal fluid; GAD, glutamic acid decarboxylase; NMDA, *N*-methyl-*D*-aspartate; VGCC, voltage-gated calcium channel; VGKC, voltage-gated potassium channel.

**Fig. 1:**
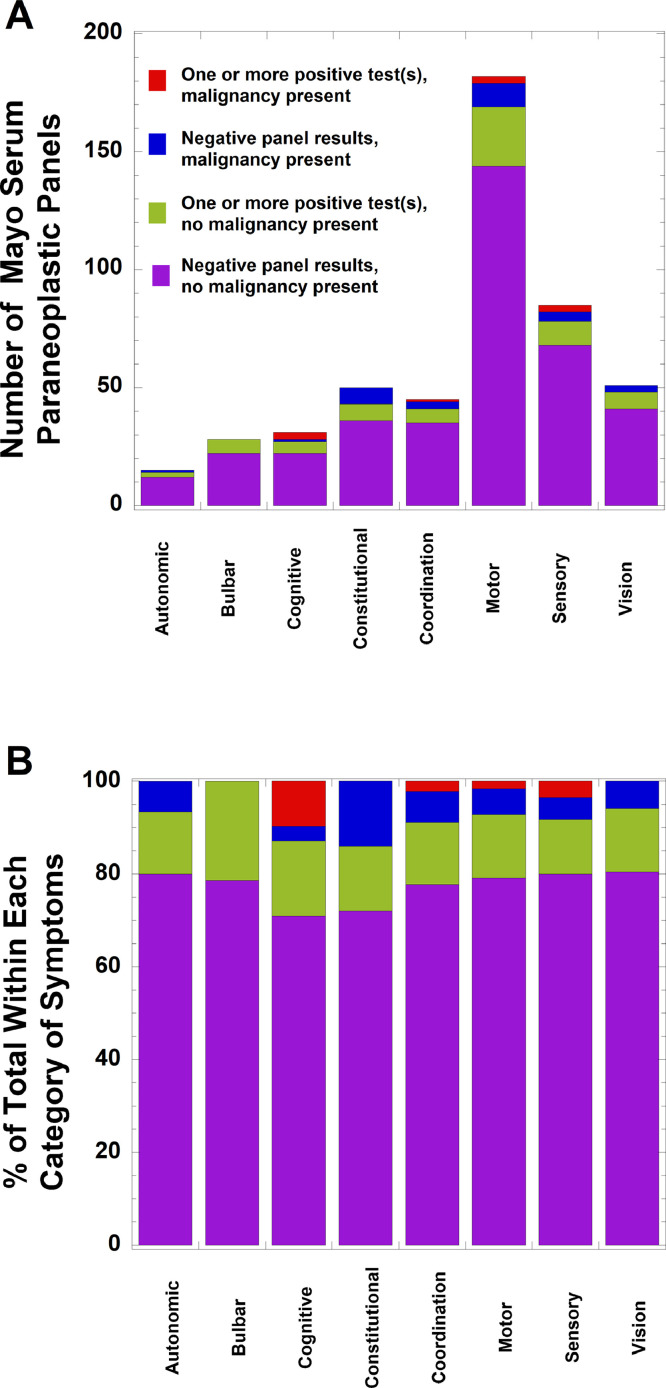
*Breakdown of testing for the Mayo serum paraneoplastic panel by category of presenting symptoms, whether patient had malignancy, and whether one or more tests on the panel were positive*. The main presenting symptoms for the patient from chart review were classified into autonomic, bulbar, cognitive, constitutional, coordination, motor, sensory, or vision. The patient category is further subdivided into four categories based on presence of malignancy and test results: (a) one or more positive results on the panel in a patient with known or later discovered malignancy (red), (b) negative results on the panel in a patient with known or later discovered malignancy (blue), (c) one or more positive results on the panel for a patient without a known malignancy (green), and (d) negative results on the panel for a patient without a known malignancy (purple). The upper panel (A) shows the data by absolute number of orders. The lower panel (B) depicts the data with a breakdown by percent within each category of presenting symptoms.

From the paraneoplastic serum panel from Mayo, autoantibodies to striational antibodies (38/483, 7.9%) and VGKC (20/483, 4.1%) were the most common detected. Fig. 1 summarizes a breakdown of testing for the Mayo serum paraneoplastic autoantibody panel, with results classified into four categories: (a) one or more positive results on the panel in a patient with known or later discovered malignancy, (b) negative results on the panel in a patient with known or later discovered malignancy, (c) one or more positive results on the panel for a patient without a known malignancy, and (d) negative results on the panel for a patient without a known malignancy. Fig. 1A shows the data by absolute number of orders. Fig. 1B depicts the data with a breakdown by percent within each category of presenting symptoms.

There was some overlap in ordering between the Mayo serum and CSF paraneoplastic panels. We identified 45 patients who had both panels ordered on the same day, with the following breakdown: 37 patients where both panels were negative, 7 patients where the Mayo serum panel had one or more positive antibodies, but the CSF panel was negative (see below for details), and 1 case where both panels showed positive Purkinje cell antibodies (titer of 1:640 in serum and 1:2048 in CSF; reference range, <1:2). The 7 patients who had one or more positive results on the Mayo serum paraneoplastic panel but not on the CSF paraneoplastic panel had the following antibodies identified in serum: striated muscle antibodies with titer of 1:120 (2 patients; reference range, < 1:60); striated muscle antibodies with titer of 1:960 (1 patient); VGKC antibodies at 0.19 nmol/L (1 patient; reference range, < 0.02); VGKC antibodies at 0.62 nmol/L (1 patient); VGKC antibodies at 0.81 nmol/L (1 patient); and striated muscle antibodies with titer of 1:64,400 (reference range < 1:60) along with acetylcholine receptor (AChR) binding antibodies at 14.9 nmol/L (1 patient; reference range <= 0.02). There were no patients for which the Mayo serum and CSF paraneoplastic panels were ordered concurrently more than once. There were also no examples where the Mayo CSF paraneoplastic antibody panel had a positive while the serum paraneoplastic antibody panel was negative. It should also be noted that AChR binding and striated muscle antibody assays were not included in the Mayo CSF paraneoplastic panel during the retrospective analysis period (Table 1).

For the ARUP serum and CSF NMDA receptor antibody tests, there were 31 unique patients who had both tests performed on the same date of collection (one patient had this happen two times for a total of 32 occurrences). In all cases except one, both tests were negative. There was a single occurrence in a 58-year-old male with constitutional symptoms of a positive CSF NMDA receptor antibody titer of 1:20 (reference range <1:1) but with serum NMDA antibody titers that were negative.

The raw data for the study are included in Supplementary files 1–7.

• Supplementary file 1: Data for 487 Mayo serum paraneoplastic panel orders (test code: PAVAL) on 483 unique patients. The retrospective timeframe is December 1, 2008 through November 30, 2018. Specific data fields include: unique patient identification number (deidentified), main category of presenting symptoms from chart review (classified into autonomic, bulbar, cognitive, constitutional, coordination, motor, sensory, or vision), patient location at time of testing (either inpatient or outpatient), sex (as recorded in the electronic medical record), age in years at time of testing, whether cancer was present at time of testing or later detected, specific cancer(s) detected (if applicable), whether there were one or more positive components on the serum paraneoplastic panel, separate columns for up to 3 positive components on the paraneoplastic panel, and whether the Mayo CSF paraneoplastic panel was ordered on same day as the serum panel and, if so, the result.
• Supplementary file 2: Data for 280 Mayo CSF paraneoplastic panel (test code: PAC1) orders on 276 unique patients. The retrospective timeframe is January 6, 2009 through November 30, 2018. Specific data fields include: unique patient identification number (deidentified), main category of presenting symptoms from chart review (classified into autonomic, bulbar, cognitive, constitutional, coordination, motor, sensory, or vision), patient location at time of testing (either inpatient or outpatient), sex (as recorded in the electronic medical record), age in years at time of testing, whether cancer was present at time of testing or later detected, specific cancer(s) detected (if applicable), whether there were one or more positives on the CSF paraneoplastic panel, whether the results for the CSF panel were available before discharge or patient death for inpatients, whether there was follow-up on the CSF panel results in the medical record, specific positives on the CSF panel (if applicable), and whether the Mayo serum paraneoplastic panel was ordered on same day as the CSF panel and, if so, the result.
• Supplementary file 3: Data for 142 ARUP Laboratories NMDA receptor antibodies in CSF (test code: 2005164) orders on 135 unique patients. The retrospective timeframe is November 19, 2012 through November 30, 2018. Specific data fields include: unique patient identification number (deidentified), category of main presenting symptoms from chart review (classified into autonomic, bulbar, cognitive, constitutional, coordination, motor, sensory, or vision), patient location at time of testing (either inpatient or outpatient), sex (as recorded in the electronic medical record), age in years at time of testing, whether cancer was present at time of testing or later detected, specific cancer(s) detected (if applicable), whether NMDA receptor CSF antibody results were positive, whether the results for the NMDA receptor CSF antibody testing were available before discharge or patient death for inpatients, whether there was follow-up on the NMDA receptor CSF antibody results in the medical record, specific results on the NMDA receptor CSF antibody testing, and whether the ARUP serum NMDA antibody was ordered on same day as the CSF NMDA receptor antibody test and, if so, the result.
• Supplementary file 4: Data for 150 ARUP Laboratories NMDA receptor antibodies in serum (test code: 2004221) orders on 144 unique patients. The retrospective timeframe is July 31, 2012 through November 30, 2018. Specific data fields include: unique patient identification number (deidentified), category of main presenting symptoms from chart review (classified into autonomic, bulbar, cognitive, constitutional, coordination, motor, sensory, or vision), patient location at time of testing (either inpatient or outpatient), sex (as recorded in the electronic medical record), age in years at time of testing, whether cancer was present at time of testing or later detected, specific cancer(s) detected (if applicable), whether NMDA receptor serum antibody results were positive, whether the results for the NMDA receptor serum antibody testing were available before discharge or patient death for inpatients, whether there was follow-up on the NMDA receptor serum antibody results in the medical record, specific results on the NMDA receptor serum antibody testing, and whether the ARUP CSF NMDA antibody was ordered on same day as the serum NMDA receptor antibody test and, if so, the result.
• Supplementary file 5: Data for 56 ARUP Laboratories P/Q-type voltage-gated calcium channel (VGCC) antibodies in serum (test code: 2004890) orders on 45 unique patients. The retrospective timeframe is July 25, 2012 through November 30, 2018. Specific data fields include: unique patient identification number (deidentified), category of main presenting symptoms from chart review (classified into autonomic, bulbar, cognitive, constitutional, coordination, motor, sensory, or vision), patient location at time of testing (either inpatient or outpatient), sex (as recorded in the electronic medical record), age in years at time of testing, whether cancer was present at time of testing or later detected, specific cancer(s) detected (if applicable), whether VGCC antibody results were positive, whether the results for the VGCC antibody testing were available before discharge or patient death for inpatients, whether there was follow-up on the VGCC antibody results in the medical record, and specific results on the VGCC serum antibody testing (reference range for testing indicated in this column).
• Supplementary file 6: Data for 110 ARUP Laboratories voltage-gated potassium channel (VGKC) antibodies in serum (test code: 3002046) orders on 107 unique patients. The retrospective timeframe is July 25, 2012 through November 30, 2018. Specific data fields include: unique patient identification number (deidentified), category of main presenting symptoms from chart review (classified into autonomic, bulbar, cognitive, constitutional, coordination, motor, sensory, or vision), patient location at time of testing (either inpatient or outpatient), sex (as recorded in the electronic medical record), age in years at time of testing, whether cancer was present at time of testing or later detected, specific cancer(s) detected (if applicable), whether VGKC antibody results were positive, whether the results for the VGKC antibody testing were available before discharge or patient death for inpatients, whether there was follow-up on the VGKC antibody results in the medical record, and specific results on the VGKC serum antibody testing (reference range for testing indicated in this column).
• Supplementary file 7: Data for 215 ARUP serum paraneoplastic reflexive panel (test code: 2013955) orders on 205 unique patients. The retrospective timeframe is October 8, 2015 through November 30, 2018. Specific data fields include: unique patient identification number (deidentified), category of main presenting symptoms from chart review (classified into autonomic, bulbar, cognitive, constitutional, coordination, motor, sensory, or vision), patient location at time of testing (either inpatient or outpatient), sex (as recorded in the electronic medical record), age in years at time of testing, whether cancer was present at time of testing or later detected, specific cancer(s) detected (if applicable), whether there were one or more positives on the ARUP serum paraneoplastic panel, whether the results for the ARUP serum paraneoplastic panel were available before discharge or patient death for inpatients, whether there was follow-up on the ARUP serum paraneoplastic panel results in the medical record, and specific positives on the ARUP serum paraneoplastic panel (if applicable).

## 2 Experimental Design, Materials and Methods

Although the overall retrospective analysis period was December 1, 2008 through November 30, 2018, some of the tests in the present study became available for ordering at various years after 2008. The details for the Supplementary Files above indicate the first date at which the particular panel or stand-alone test was available at our medical center. All data was obtained from patient data in the electronic medical record from the University of Iowa Hospitals and Clinics (Iowa City, Iowa, United States). A reporting tool within the electronic medical record, known as Epic Reporting Workbench, was used to identify all tests in Table 2 performed in the retrospective timeframe. Only data from patients who had the paraneoplastic tests or test panels described in the present study performed at the University of Iowa Hospitals and Clinics were included; no data was obtained from diagnostic vendors or reference laboratory databases for any of the laboratory assays used for clinical testing. Detailed chart review was performed on all results, regardless of whether the particular test was positive or negative. For classification of presenting symptoms, we followed the categories utilized by Alabareen et al. (autonomic, bulbar, cognitive, constitutional, coordination, motor, sensory, or vision) in their retrospective analysis of patients tested by the Mayo paraneoplastic autoantibody panel [8].

## Ethics Statement

The analyses had approval by the University of Iowa Institutional Review Board (protocol # 201812702) as a retrospective project.

## CRediT authorship contribution statement

**Matthew D. Krasowski:** Formal analysis, Conceptualization, Writing – original draft, Writing – review & editing, Methodology, Supervision. **Anna Dolezal:** Formal analysis, Writing – original draft, Writing – review & editing. **Bryan W. Steussy:** Formal analysis, Writing – review & editing. **Michael P. Gailey:** Formal analysis, Writing – review & editing. **Benjamin W. Darbro:** Formal analysis, Conceptualization, Writing – review & editing, Methodology, Supervision.

## Declaration of Competing Interest

The authors declare that they have no known competing financial interests or personal relationships that could have appeared to influence the work reported in this paper.

## References

[1] Berger B., Bischler P., Dersch R., Hottenrott T., Rauer S., Stich O. (2015). ``Non-classical'' paraneoplastic neurological syndromes associated with well-characterized antineuronal antibodies as compared to ``classical'' syndromes-more frequent than expected. J. Neurol. Sci..

[2] Darnell R.B., Posner J.B. (2003). Paraneoplastic syndromes involving the nervous system. N. Engl. J. Med..

[3] Yanagihashi M., Kawabe K., Ikeda K. (2013). Presence of paraneoplastic antibodies in non-carcinomatous patients with neurological involvements of unknown cause. J. Neurol. Sci..

[4] Budhram A., Dubey D., Sechi E., Flanagan E.P., Yang L., Bhayana V., McKeon A., Pittock S.J., Mills J.R. (2020). Neural antibody testing in patients with suspected autoimmune encephalitis. Clin. Chem..

[5] Horta E.S., Lennon V.A., Lachance D.H., Jenkins S.M., Smith C.Y., McKeon A., Klein C., Pittock S.J. (2014). Neural autoantibody clusters aid diagnosis of cancer. Clin. Cancer Res..

[6] Tebo A.E., Haven T.R., Jackson B.R. (2016). Autoantibody diversity in paraneoplastic syndromes and related disorders: the need for a more guided screening approach. Clin. Chim. Acta.

[7] Zoccarato M., Gastaldi M., Zuliani L., Biagioli T., Brogi M., Bernardi G., Corsini E., Bazzigaluppi E., Fazio R., Giannotta C. (2017). Diagnostics of paraneoplastic neurological syndromes. Neurol. Sci..

[8] Albadareen R., Gronseth G., Goeden M., Sharrock M., Lechtenberg C., Wang Y. (2017). Paraneoplastic autoantibody panels: sensitivity and specificity, a retrospective cohort. Int. J. Neurosci..

[9] Callaghan B.C., Burke J.F. (2019). Author response: Unintended consequences of Mayo paraneoplastic evaluations. Neurology.

[10] Ebright M.J., Li S.H., Reynolds E., Burke J.F., Claytor B.R., Grisold A., Banerjee M., Callaghan B.C. (2018). Unintended consequences of Mayo paraneoplastic evaluations. Neurology.

[11] Kelkar P. (2019). Reader response: unintended consequences of Mayo paraneoplastic evaluations. Neurology.

[12] Kim J.T., Harris N.S. (2019). Utilization review of paraneoplastic neurological syndrome antibody screening panels: experience at a tertiary academic health center. J. Appl. Lab. Med..

[13] Zidan A., Fein A., Zuchowski K. (2019). The use, misuse and abuse of paraneoplastic panels in neurological disorders. A retrospective study. Clin. Neurol. Neurosurg..

[14] (2019). Hermes Tacker D: Clinical utility and performance in test utilization interventions for paraneoplastic antibody panel orders: a delicate balance. J. Appl. Lab. Med..

[15] Pittock S.J., Mills J.R., McKeon A. (2019). Reader response: Unintended consequences of Mayo paraneoplastic evaluations. Neurology.

[16] Seluk L., Taliansky A., Yonath H., Gilburd B., Amital H., Shoenfeld Y., Kivity S. (2019). A large screen for paraneoplastic neurological autoantibodies; diagnosis and predictive values. Clin. Immunol..

[17] Trevisiol C., Cani I., Fabricio A.S.C., Gion M., Giometto B., De Massis P. (2020). Serum tumor markers in paraneoplastic neurologic syndromes: a systematic review of guidelines. Front. Neurol..

